# HTR8/SVneo Cells Display Trophoblast Progenitor Cell-Like Characteristics Indicative of Self-Renewal, Repopulation Activity, and Expression of “Stemness-” Associated Transcription Factors

**DOI:** 10.1155/2013/243649

**Published:** 2013-03-19

**Authors:** Maja Weber, Ilka Knoefler, Ekkehard Schleussner, Udo R. Markert, Justine S. Fitzgerald

**Affiliations:** ^1^Placenta-Lab, Department of Obstetrics, University Hospital Jena, Germany; ^2^Abteilung für Geburtshilfe, Placenta-Labor, Universitätsklinikum Jena, Bachstr. 18, 07740 Jena, Germany

## Abstract

*Introduction*. JEG3 is a choriocarcinoma—and HTR8/SVneo a transformed extravillous trophoblast—cell line often used to model the physiologically invasive extravillous trophoblast. Past studies suggest that these cell lines possess some stem or progenitor cell characteristics. Aim was to study whether these cells fulfill minimum criteria used to identify stem-like (progenitor) cells. In summary, we found that the expression profile of HTR8/SVneo (CDX2+, NOTCH1+, SOX2+, NANOG+, and OCT-) is distinct from JEG3 (CDX2+ and NOTCH1+) as seen only in human-serum blocked immunocytochemistry. This correlates with HTR8/SVneo's self-renewal capacities, as made visible via spheroid formation and multi-passagability in hanging drops protocols paralleling those used to maintain embryoid bodies. JEG3 displayed only low propensity to form and reform spheroids. HTR8/SVneo spheroids migrated to cover and seemingly repopulate human chorionic villi during confrontation cultures with placental explants in hanging drops. We conclude that HTR8/SVneo spheroid cells possess progenitor cell traits that are probably attained through corruption of “stemness-” associated transcription factor networks. Furthermore, trophoblastic cells are highly prone to unspecific binding, which is resistant to conventional blocking methods, but which can be alleviated through blockage with human serum.

## 1. Introduction

The master regulatory networks of human embryonic stem cell (hESC) transcription factors, OCT4, SOX2, and NANOG, as well as other cell fate determining transcription factors that are implicated in stem cell self-renewal capacities, such as NOTCH1 and STAT3, are expressed not only by embryonic stem cells, but also by a number of cancers [1]. Some of these factors are also expressed in choriocarcinoma (gestational trophoblastic disease) [2]. This has led to the thought that choriocarcinoma may also represent a group of tumors, in which hESC transcription factor deregulation has led to their transformation into cancer stem cells. 

In mammalian development, the first cell differentiation step segregates trophoblast and embryonic cell lineages, thus resulting in the formation of the blastocyst's outer lining, the trophectoderm (TE), and its inner cell mass (ICM). The trophectoderm consists of trophoblast stem cells that express CDX2, a homeobox transcription factor, which is required for the emergence of these cells [3]. Physiological invasion is seen during blastocyst implantation, which is mediated through the trophectoderm. Interestingly, both CDX2 and SOX2 deficiency lead to implantation failure of the blastocyst secondary to trophoectoderm differentiation problems [4–6]. 

The trophectoderm also differentiates into several trophoblast subsets in order to create the placenta of the first trimester pregnancy. Of these subsets, the cytotrophoblast is considered a putative “progenitor cell,” which replenishes the outer layer of the villous (syncytiotrophoblast), but which is also able to invade the decidua in a cancer-like manner when necessary and desirable (extravillous trophoblast) [7]. This behaviour is often believed to be driven by hypoxia, and it is a well-orchestrated and closely controlled process, mostly through a network of interaction between the invading trophoblast, the decidua, the maternal endothelium, and the maternal immune system; the detailed description of which would tax the scope of this introduction [8]. The first trimester placenta is especially ample with invasive (cyto)trophoblast, while the term placenta trophoblast loses this capability [8]. 

The uniqueness of this situation, in which physiologic, spatially (limited to the decidua, first third of the myometrium, and the invasion into maternal spiral arteries), and temporally (limited to the first trimester of pregnancy) regulated invasion (by the trophoblast) and pathologic, de-regulated, and malignant invasion (by choriocarcinoma) are set so close together, has drawn the attention of cancer researchers worldwide [8]. However, since isolation of primary trophoblast and choriocarcinoma cells is often cumbersome, in recent years, several trophoblastic cell lines have been utilized as imperfect models for the invasive trophoblast(ic) cell. Some of the most popular cell lines used constitute the immortalized first trimester trophoblast cell line, HTR8/SVneo, and the choriocarcinoma cell line JEG3. HTR8/SVneo cells are often considered a closer model of trophoblast cells, because the HTR8/SVneo cell lines were established by immortalizing a physiologic extravillous trophoblast cell via transfection with a plasmid containing the simian virus 40 large T antigen (SV40) [9], while the JEG3 cell line was cloned from a primary choriocarcinoma strain [10]. 

Our own recently published data, however, demonstrate that the miRNA profiles of these two cell lines are quite differing, surprisingly with JEG3 encompassing an miRNA profile that is closer to primary first trimester trophoblast cells than that of the HTR8/SVneo cell lines [11]. Villous cytotrophoblast and HTR8/SVneo cells have interestingly also been implicated in producing a “side population” that either demonstrates long-term repopulating properties or expresses classical hESC markers [12, 13]. 

Following the idea that both JEG3 and HTR8/SVneo are transformed cells and have been proposed to produce cancer stem cell or progenitor (side population) cell populations, we aimed to characterize the putative cancer and trophoblast stem/progenitor cell traits of HTR8/SVneo and JEG3 cells on the basis of general minimum recommendations for identifying cancer stem cells or progenitor cells [14, 15]. This is accomplished first by assessing the capacity of these cells to form spheroid bodies, second by determining the expression of various transcription factors related to progenitor or to cancer stem cell development, and finally by investigating the cells' ability to repopulate trophoblast tissue in a near in vivo model. 

For these studies, we phrase SOX2, OCT4, and NANOG as core “stemness-” associated transcription factors [16] and CDX2 [3] as a trophoblast stem/progenitor cell transcription factor. NOTCH1 is included as an often abused, prominent cell-fate transcription factor associated with both cancer stem cells and hESC [17–20] and is, henceforth, termed a cell-fate determining transcription factor.

## 2. Methods

### 2.1. Spheroid Formation with Hanging Drops

We chose the hanging drops protocol as reviewed by Kurosawa [21].

**Figure 1:**
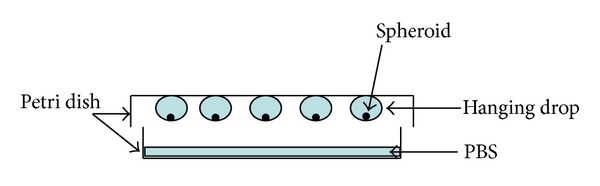
Schematic diagram of spheroid formation.

Briefly, 20 000 cells per 30 *μ*L drop supplemented RPMI (as described later in Section 2.2) were plated onto the lid of two Petri dishes in regular arrays (20 drops/Petri lid). The lid was inverted over the bottom of the PBS-filled Petri dish (see schematic representation Figure 1). The Petri dish with the hanging drops were cultured under standard conditions (37°C, 5% CO_2_, humidified atmosphere) for 48 hours. A schematic image of the hanging drop principle is seen in Figure 1. The experiment was carried out in the same manner for HTR8/SVneo cells and for JEG3 cells (40 drops per cell line).

### 2.2. Cell Culture

HTR-8/SVneo cells (a kind gift from Professor Charles Graham of the Department of Anatomy and Cell Biology at Queen's University, Kingston, ON, Canada) were cultured in RPMI (PAA) and JEG3 cells in F12 Medium. Both media were supplemented with 10% fetal bovine serum (FBS; SIGMA, St. Louis, USA) and 1x penicillin/streptomycin (PAA Laboratories; Pasching, Austria). Cell cultures were maintained under standard culturing conditions (37°C, 5% CO_2_, humidified atmosphere).

### 2.3. Immunocytochemistry (ICH)

Cells were trypsinized, centrifuged, and resuspended in 1 mL respective medium. Slides (SuperFrost/Plus slides; Menzel, Germany) were washed and sterilized with ethanol, coated with cells (200 *μ*L), and incubated over night at 37°C. The cells were fixed on the next day with ethanol/methanol and consequently used to perform immunocytochemistry. To inhibit endogenous peroxidise activity, the cells were incubated in methanol/H_2_O_2_ for 5–10 min and washed for 5 min in phosphate-buffered saline (PBS, pH 7.4), followed by incubation firstly with and without 5% human AB serum (PAA), which corresponds to an approximate Fc-concentration of 0.6 mg/mL, in order to further eliminate the possibility of Fc-receptor cross-reactions (as described in [22]), and secondly with goat serum at room temperature for 20 min (Vector Laboratories) to eliminate regular nonspecific background staining. 

Samples were then incubated with the primary antibodies (please refer to Table 1) for 60 min at room temperature. Antibodies were diluted in DAKO Antibody Diluent with Background Reducing Components (DAKO, Denmark). In the next step, our samples were incubated with the biotinylated secondary antibody (Vector Laboratories) for 30 min at room temperature. For a listing of antibodies, please refer also to Table 1. Following incubation with the secondary antibody, an incubation period with ABC-complex (avidin-biotinylated peroxidise; Vector Laboratories) again for 30 min at room temperature was completed. Between each step, all samples were washed profusely with PBS. The peroxidase reaction was achieved with DAB (diaminobenzidine/H_2_O_2_; 1 mg/mL; DAB; Dako) and after 5 min discontinued with water. Hematoxylin staining was used for cell nuclei staining (2 min). Finally, slides were dehydrated by an ethanol-to-xylene treatment, covered with Histofluid (Paul Marienfeld, Lauda-Königshofen, Germany), and analysis was completed with the Axioplan 2 microscope (Carl Zeiss, Jena, Germany).

A negative control was prepared by replacing the primary antibody with DAKO Antibody Diluent only. Isotype controls were prepared in the same manner as the primary antibody.

Analysis of staining intensity and gross estimation of stained cell numbers was accomplished by eye and by two blinded investigators (criteria similar to standard immunoreactive scoring).

### 2.4. Immunofluorescence Staining

The cells were cultured on SuperFrost/Plus slides (Menzel, Germany) over night with serum-free media. Cells were fixed on the subsequent day with ethanol/methanol. To reduce nonspecific background staining, all samples were incubated either with goat serum or with 5% human AB serum (PAA, as recommended by [22]) at room temperature for 20 min (Vector Laboratories). Samples were incubated with the primary antibodies (please refer to Table 1 for company names and concentrations used) overnight at 4°C. Antibodies were diluted in DAKO Antibody Diluent with Background Reducing Components (DAKO, Denmark). On the next day, they were incubated with the secondary antibody labeled with Cy3 for 1 h at room temperature. Between each step, all samples were washed profusely with PBS. Sections were counterstained Vectashield Mounting Media with DAPI (VECTOR Laboratories) and then cover slipped. 

A negative control was prepared by replacing the primary antibody with DAKO Antibody Diluent only. Isotype controls were prepared in the same manner as the primary antibody. All samples were analyzed with an AxioPlan2 microscope (Carl Zeiss, Jena, Germany). 

Assessment was accomplished by eye and by two investigators only in terms of positive or negative expression and pattern of expression.

### 2.5. Placental Explant Cultures and Confrontation Cultures

Two biopsy-sized “explants” (2 mm diameter) each from villous tissue of five healthy human term placentae (after elective caesarian section) were collected. An approval by the local ethical committee exists. Prior to confrontation cultures, all spheroids were stained by application of 10 nM Mito Tracker dye (fluorescent green; Invitrogen) for 30 min at 37°C and then intensively washed in PBS (Biochrom, Germany). Commencing from the time that the placental “biopsies” or “explants” are placed in culture, these are termed villous explant cultures. 

Each explant was then confronted in culture with one spheroid within the respective hanging drop for 48 h. Subsequently, the confronted tissues were incubated with a solution of 10% nonfat milk in PBS containing 1% Triton (AppliChem) to permeabilize cell membranes and to block nonspecific binding sites for 1 h. Following intensive washing step, tissues were incubated with a rat anti-human CD31 (anti-PECAM1; Millipore, Germany) for 2 h, followed by incubation with a goat anti-rat IgG-Cy5 conjugate (Milipore) for 90 min, all within the previously described solution (nonfat milk/PBS/Triton). After staining, descriptive analyses with the tissues and spheroids were accomplished on a confocal laser scanning microscope (Carl-Zeiss, Jena, Germany).

## 3. Results

### 3.1. HTR8/SVneo Cells Have a High and JEG3 Cells a Low Propensity to Form Spheroid Bodies Within Hanging Drops

The first step to confirm putative cancer stem/progenitor cell status is to confirm their capacity for self-renewal, which is often accomplished by propagation of these cells as spheroids in stem cell culturing conditions [14]. Cells with self-renewing potential can be disaggregated from the spheroids and passaged multiple times with retention of spheroid-forming ability [15].

**Figure 2:**
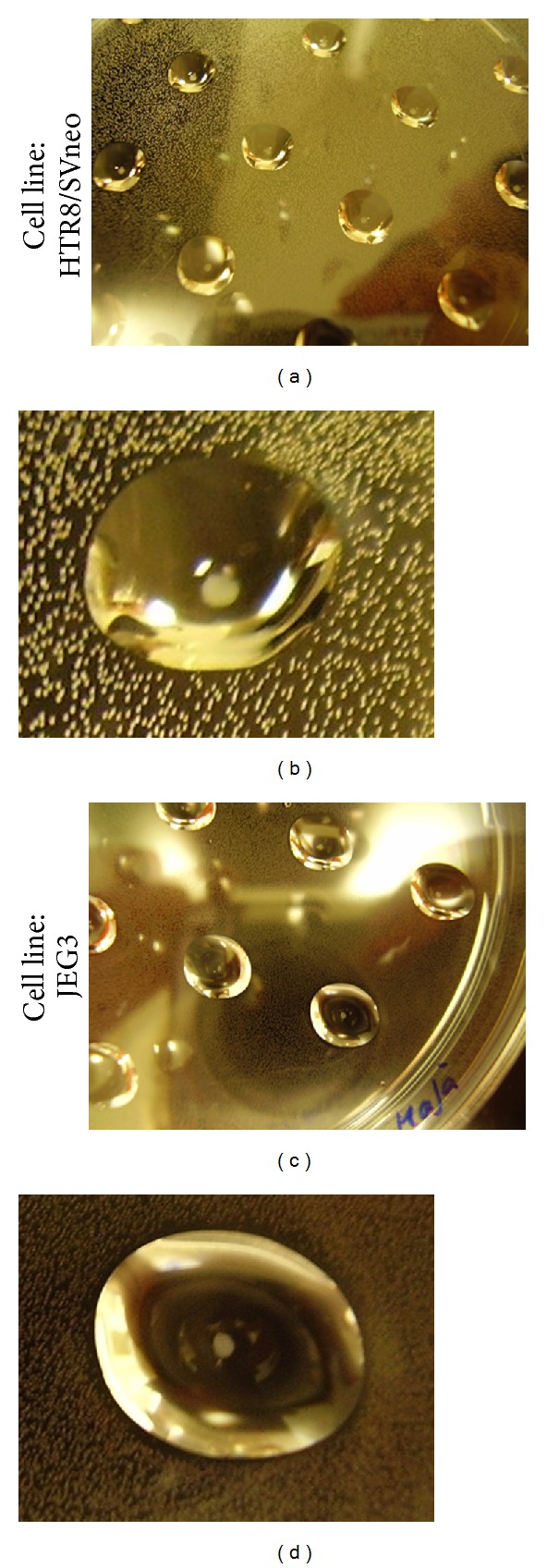
Spheroid formation of HTR8/SVneo and JEG3 cells via hanging drops. Forty hanging drops per cell line were produced with 20 000 cells per 30 *μ*L drop. The environment of the hanging drop delivers the prerequisite for spheroid formation. After an incubation period of 48 h, 100% of HTR8/SVneo-containing drops formed visible spheroids (a, b), while only 50% of JEG3-containing drops formed spheroids (c). JEG3 spheroids appeared smaller in circumference, less globular (oblong shapes), and rather on the verge of disaggregation (d).

Of the 40 hanging drops experiments per cell line, 100% of the incubated HTR8/SVneo cells and only 50% of the incubated JEG3 cells were able to form spheroids (data not demonstrated). The developed HTR8/SVneo spheroids regularly measured a diameter of approximately 700–750 *μ*m, which was also visible by the “naked” eye (Figures 2(a) and 2(b)). On the other hand, JEG3 spheroids were much smaller (as visible even by the “naked” eye) and irregularly shaped (Figures 2(c) and 2(d)). The JEG3 spheroids were also unstable and were disaggregated easily and thus could not easily be pipetted into the shortened tip of a pipette for transportation into, for example, a new hanging drop.

In order to verify that spheroid formation is not secondary to cell aggregation, we proceeded to disaggregate the spheroids, split these cells, and propagate them again in hanging drops to assess their continued ability to reform spheroids (as recommended in [15]). Under this experimental setting, we passaged the HTR8/SVneo spheroids multiple times (5 passages and continuing) without their loss of spheroid-forming abilities. As little as 5000 HTR8/SVneo cells are able to form a spheroid (splitting ongoing). Please note that we have not yet tried to form spheroids with less cells than the mentioned. Only 50% of the initial JEG3 spheroids were able to reform spheroids following disaggregation and splitting; further passages could not be maintained.

### 3.2. HTR8/SVneo and JEG3 Cells Express the Trophoblast Stem Cell Marker CDX2

CDX2 is a transcription factor that is necessary for the first differentiation of hESC into the trophoblast stem cell (reviewed in [3]). Loss of CDX2 activity disables the blastocyst to implant correctly during murine pregnancy [23]. Following the hypothesis that HTR8/SVneo or JEG3 cells recapitulate features of a cancer stem cell or a trophoblast(ic) stem or progenitor cell, we sought to investigate whether the classic trophoblast stem cell “marker” is expressed in both cell lines.

Since it has been described that nonspecific binding in trophoblast(ic) cells is extreme and often cannot be alleviated through conventional blocking procedures [22], we initially blocked cell lines with normal goat serum and further blocked them with or without human serum. According to immunofluorescence staining results, both cell lines seem to stain positive for CDX2. However, since there was no alteration in the pattern of staining before and after application of human serum, and since it is rather unlikely that CDX2 is so prominently expressed in the cytoplasm [24, 25], we conclude that unspecific binding is still too high in immunofluorescence stainings to be reliable. 

**Figure 3:**
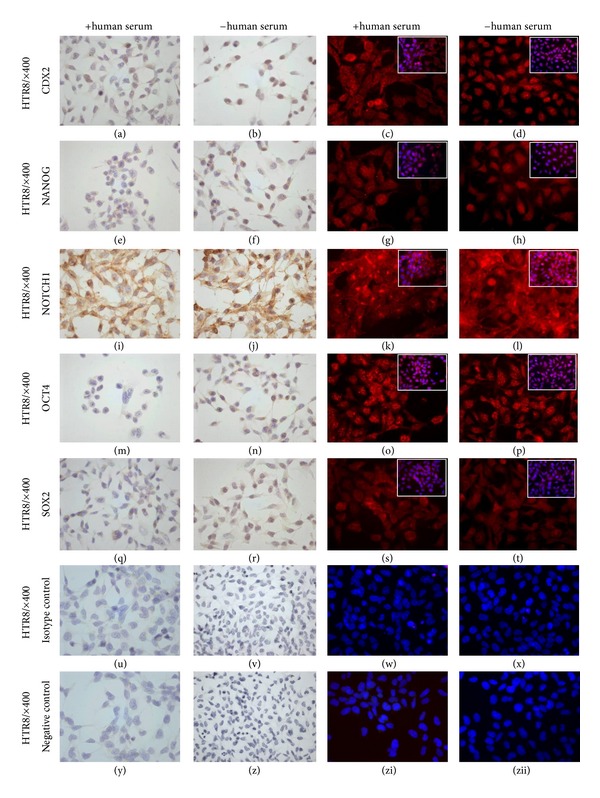
Expression of trophoblast (CDX2), cell fate determining (NOTCH1), and core “stemness-” associated (OCT4, NANOG, and SOX2) stem cell transcription factors in HTR8/SVneo cells. HTR8/SVneo cells express CDX2, NOTCH1, NANOG, and SOX2 ((a), (e), (i), and (q)). OCT4 signaling is lost after blocking the samples with human serum ((m) versus (n)). Human serum is needed to further block exceptional, unspecific binding on trophoblastic cells that cannot be eliminated via conventional blocking measures. Immunofluorescent staining seems less specific with or without blockage with human serum ((c), (d), (g), (h), (k), (l), (o), (p), (s), and (t)).

**Figure 4:**
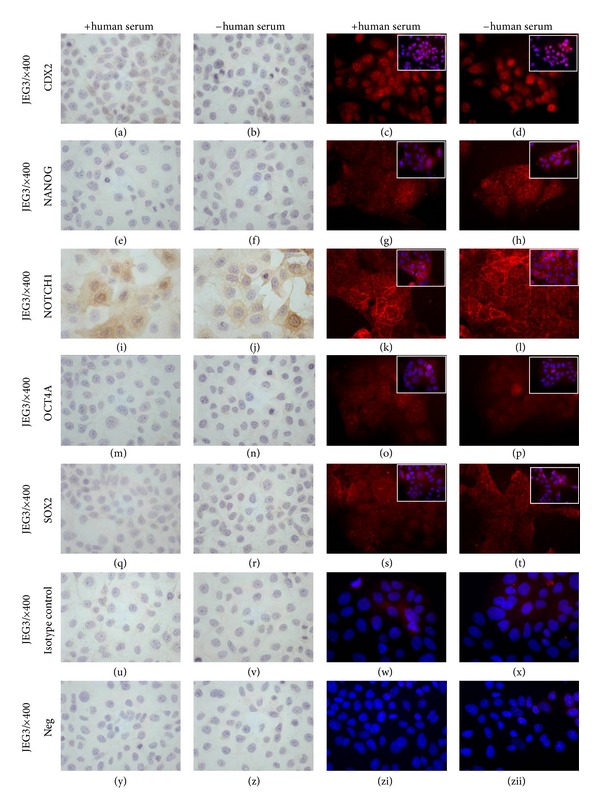
Expression of trophoblast (CDX2), cell stypo determining (NOTCH1), and core “stemmness-” associated (OCT4, NANOG, and SOX2) stem cell transcription factors in JEG3 cells. JEG3 cells express CDX2 and NOTCH1 ((a) and (i)). OCT4, NANOG and SOX2 signaling are not visible (with human serum: (e), (m), and (q); without human serum (f), (n), and (r)). Immunofluorescent staining seems less specific with or without blockage with human serum (all positive signals: (c), (d), (g), (h), (k), (l), (o), (p), (s), and (t)).

In contrast, ICH results show that HTR8/SVneo cells express CDX2 in the nucleus, although the trophoblast marker expression appeared lower, especially in terms of number of nuclei, after blockage with human serum (Figure 3(b) without human serum versus Figure 3(a) with human serum). In JEG3 cells, a low-intensity CDX2 signal was observable even after blocking with human serum with numbers of positive-stained nuclei rather unchanged (Figures 4(a) and 4(b)). Via immunofluorescence staining procedures, CDX2 expression is made visible, and the signal patterns are again not affected by blocking procedure; thus, this procedure is again deemed unreliable (Figures 3(c), 3(d), 4(c), and 4(d)). 

### 3.3. HTR8/SVneo Cells Express NOTCH1, NANOG, and SOX2, but Not OCT4 according to ICH Analysis

In theory, cancer stem cells have probably reacquired properties similar to stem cells during transformation into a malignancy [1, 2, 26]. We analyzed the expression of the classic or core “stemness-” associated transcription factors OCT4, NANOG, and SOX2 (reviewed in [16]). We also chose to assess the expression of the cell fate determining transcription factor NOTCH1 due to its recent suggestion as a maintainer of “stemness,” as well its ever-emerging role in the invasion potential of tumors (reviewed in [17, 20, 27, 28]).

Since it has been described that nonspecific binding in trophoblast(ic) cells is extreme and often cannot be alleviated through conventional blocking procedures [22], we initially blocked cell lines with normal goat serum and further blocked them with or without human serum. Upon blocking of HTR8/SVneo cells with human serum, the positive signals for all investigated transcription factors were reduced, especially in the nuclei, or, as in the case of OCT4a, disappeared altogether (Figure 3: (f), (j), (n), and (r) without human serum versus (e), (i), (m), and (q) with human serum). The expression intensity was highest for NOTCH1, and this expression was only slightly altered after blockage with human serum (Figures 3(i) and 3(j)). In contrast to peroxidase staining, we detected all stem cell markers via fluorescence staining, and there were no visible differences in staining pattern between the blocking procedures (Figure 3, for NANOG: (g) and (h); for NOTCH1: (k) and (l); for OCT4: (o) and (p); for SOX2: (s) and (t)).

Taking the recommendations of Honig et al. [22] into consideration (meaning that we now deem the immunofluorescence procedure unreliable for staining of these transcription factors in these cells), we surmise that HTR8/SVneo cells express all of the investigated transcription factors except for OCT4. 

### 3.4. JEG3 Cells Express NOTCH1, but Not NANOG, SOX2, and OCT4 according to ICH Analysis

NANOG, OCT4, and SOX2 were not detectable in JEG3 cells regardless of blocking methods (Figure 4: (e), (f), (m), (n), (q), and (r)) after ICH analysis. 

JEG3 cells express NOTCH1, and, as in HTR8/SVneo cells, NOTCH1 was detectable with both blocking methods (Figures 4(i) and 4(j)). Interestingly, the NOTCH1 staining signal in JEG3 cells appears less intensive than that in HTR8/SVneo cells (Figure 3(i) versus Figure 4(i)).

Similar to HTR8/SVneo cells, the fluorescent staining of JEG3 cells shows positive signals for NANOG, NOTCH1, OCT4, and SOX2, and it makes no difference in staining pattern if the cells were blocked with or without human serum (Figure 4: (g), (h), (k), (l), (o), (p), (s), and (t)). 

### 3.5. HTR8/SVneo Spheroids Contain Chorionic Villi-Covering Cells Indicative of Repopulation

To demonstrate repopulation capacity of cancer stem/progenitor cells, the progenitor cell candidates are usually injected in an immunocompromised mouse in the general vicinity of the target organ in which prior elimination of the target population has taken place (as the progenitor cells are supposed to replace them) [29]. If the progenitor cell candidates (e.g., mammary gland stem cell) possess repopulating potential, then they will be found in the stead of the original target cell population (e.g., mammary gland).

It has been observed before that altering in vitro culture standards can cause a side population of HTR8/SVneo cells to differentiate into trophoblast subpopulations, as made visible by ICH analysis of differentiation markers [12]. The HTR8/SVneo capacity to actually repopulate the villous in an in vivo setting has not yet been demonstrated. 

The syncytiotrophoblast layer within villous explant cultures is known to fully degenerate following 4 h of culture initiation [30].

We sought to determine whether the cells present in the HTR8/SVneo spheroids could repopulate the entire chorionic villous, which according to the previous citation is now replete of the syncytiotrophoblast layer, following confrontation with placental villous explants in an effort to remain in a near in vivo model of the placenta. 

**Figure 5:**
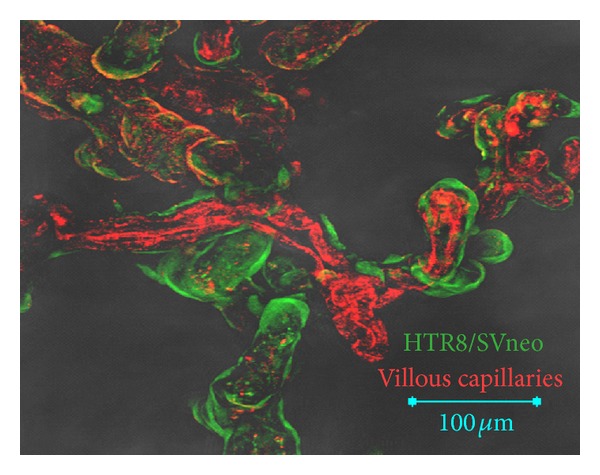
HTR8/SVneo spheroid cells repopulate chorionic villi of placental biopsies. HTR8/SVneo spheroids were confronted with placental biopsies derived from healthy pregnancies after elective, ceasarian section. After 48 h, HTR8/SVneo spheroid cells (green) have covered chorionic villi (endothelium of fetal capillaries within the villi are depicted in red).

We excluded JEG3 from this experiment as the previous investigations suggest that JEG3 cells possess only low prominent cancer stem cell characteristics. Furthermore, as JEG3 spheroids were unstable and easily disaggregated, it was not possible to transfer them to new hanging drops in which coculture experiments were performed. In coculture of HTR8/SVneo with placental explants, spheroids are completely disaggregated after 48 h, and all retrievable HTR8/SVneo cells cover the chorionic villous (Figure 5; HTR8/SVneo spheroid cells in green; endothelium of fetal capillaries within chorionic villi in red). Ten individual confrontation cultures have been performed with qualitatively similar results. 

Assuming that the syncytiotrophoblast layer of placental explants truly do degenerate following 4 h cultivation, then the previous situation suggests that the HTR8/SVneo spheroid cells contain the progenitor cell characteristic of repopulating activity in a near in vivo model. 

## 4. Discussion

HTR8/SVneo and JEG3 cells are highly popular transformed cell lines often used as an imperfect model of the trophoblast, with HTR8/SVneo often deemed as closer to the physiologic setting. Both cell types have been proposed to possess “stem-like” or “progenitor-like” characteristics [2, 12]. 

One aim of this study was to characterize stem/progenitor cell traits of these cell lines according to general minimum standards used to identify putative cancer stem cells [14, 15]. Another aim of this study was to characterize the HTR8/SVneo and JEG3 cell lines for their expression pattern of various transcription factors associated with “stemness” (OCT4, NANOG, and SOX2), cell fate (NOTCH1), and trophoblast stem cells (CDX2) and to correlate this to their intrinsic progenitor/stem cell capacities, because their transformed character is likely to contribute to altered transcription factor expression, which in turn is likely to be responsible for stem/progenitor-like behavior. This is a first step in defining a cell population as a progenitor or cancer stem cell. 

In our hands, HTR8/SVneo cells have a high propensity to form spheroid bodies, while expressing virtually all investigated transcription factors (specifically CDX2, NOTCH1, SOX2, and NANOG, and not OCT4). Furthermore, HTR8/SVneo spheroid cells demonstrate behavior reminiscent of self-renewal and replenishing properties. In contrast, JEG3 cells have only a limited ability to form spheroid bodies, and although they express the trophoblast stem cell marker CDX2 and a cell fate determinant NOTCH1, they did not express the investigated hESC cell markers or so-called “stemness-” associated transcription factors. Taken together, this is indicative of the fact that HTR8/SVneo cells are transformed in a manner that might make them closer in phenotype to a trophoblast progenitor cell than to a differentiated trophoblast cell, such as a syncytio- or extravillous trophoblast. Our experiments reveal traits that speak for JEG3 cells being a form of cancer stem cell as previously proposed. 

As mentioned earlier, our own recent results indicate that HTR8/SVneo cells have less in common with the primary extravillous trophoblast cell than JEG3 cells [11]. GeneChip analyses of the expression signatures of primary trophoblast versus choriocarcinoma cell lines (including JEG3) and versus extravillous trophoblast derived cell lines (including HTR8/SVneo) have revealed that all three groups cluster distinctly [31]. Furthermore, the genes that are similarly expressed between EVT and choriocarcinoma cell lines are related to cell motility, signaling, vasculature and tissue development (all functional signs of differentiation), while HTR8/SVneo and EVT similarities are restricted to those genes regulating RNA transport and metabolism (housekeeping characteristics) [31]. Classic hallmark characteristics for primary, differentiated trophoblast cells are its expression of cytokeratin 7 and negative expression of vimentin [32, 33]. HTR8/SVneo cells show an expression profile that is just the opposite [12, 31]. In this aspect, it is interesting that HTR8/SVneo cells express N-cadherin, while their JEG3 counterparts do not [34], meaning that HTR8/SVneo express at least two known epithelial-mesenchymal transition (EMT) markers (N-cadherin and vimentin), which is also a sign of partial dedifferentiation (reviewed in [27]). Finally, HTR8/SVneo cells express HLA-G, a typical extravillous trophoblast differentiation marker [33], only weakly or not at all [12] and secrete *β*-HCG, a hallmark characteristic of a syncytiotrophoblast cell (as reviewed in [35]), weakly, but more than primary cytotrophoblast, which do not secrete hCG at all [9]. 

In our study, we also demonstrate the self-renewal properties of HTR8/SVneo cells propagated as spheroids under the same culture conditions as human embryonic stem cell embryoid bodies. HTR8/SVneo cells not only survive multipassages after spheroid formation, but are continually able to reform spheroids after each passage even with fairly low cell numbers. This is also in line with a recent observation that HTR8/SVneo cells form a side population that displays self-renewal characteristics [12]. We found it somewhat puzzling that all HTR8/SVneo cells incubated in hanging drops formed spheroids in our experiments, while Takao et al. describe only a very exclusive side-population that demonstrates self-renewal properties [12]. However, since we excluded the possibility that HTR8/SVneo spheroids are mere cell aggregates, we propose that the hanging drop environment induces HTR8/SVneo cells to alter its phenotype towards that of a progenitor-like cell.

We have not proven in our own studies that the syncytiotrophoblast layer of placental explants has actually degenerated during the 48 h cultivation period we chose; however, other studies have described that this occurs after 4 h [30]. Following coculture of our placental explants with HTR8/SVneo cells for 48 h, we reveal for the first time that HTR8/SVneo cells have the propensity to “renew” or at least cover villous tissue in a model near to the in vivo situation. We speculate that the syncytiotrophoblast layer is lost, as described, and that HTR8/SVneo cells answer a distress call during the cultivation period, which allows the HTR8/SVneo cells to migrate to this area and cover or indeed replace the villous with syncytiotrophoblast-like cells. This is an indication for a certain degree of plasticity within the HTR8/SVneo cells, which we have not corroborated here with syncytiotrophoblast-specific expression of surface molecules. We believe that this is likely though, since Takao et al. have been able to demonstrate that a side-population of HTR8/SVneo cells is able to differentiate into syncytiotrophoblast and other trophoblast lineages [12]. Future studies are needed in order to unravel which transcription factor or other signal is responsible for regulating this migratory and replenishing characteristic. 

Our investigations characterize for the first time the expression of “stemness-” associated cancer and trophoblast stem cell transcription factors in the HTR8/SVneo cell line. The fact that both cell lines produce CDX2, while only HTR8/SVneo shows progenitor cell capacities, could indicate that CDX2 is rather a sign of trophoblast lineage derivation instead of a trophoblast stem cell differentiation alone. However, CDX2 has been shown to be expressed only in first trimester trophoblast, while term placentae lose this capacity. Furthermore, the same study identified that CDX2+ELF5+ cells within the placentae characterize cytotrophoblast populations [36]. This in turn suggests that JEG3 cells have retained a certain cytotrophoblastic identity, which we have not been able to visualize as a progenitor-like function in this study.

The regulatory network between CDX2 and the “stemness-” associated transcription factors is tight and complicated; reiterating this in detail goes beyond the scope of this investigation. Briefly, CDX2 and the “stemness-” associated transcription factors are thought to reciprocate or mutually antagonize each other, also because this is the expression pattern found during blastocyst formation. At least in the mouse, CDX2 is known to downregulate OCT4 and NANOG [37, 38]. However, SOX2 and CDX2 are known to cooperate during trophectoderm formation [4]. In our analysis, while JEG3 cells did not express any of the so-called “stemness-” associated transcription markers, HTR8/SVneo cells expressed most of them (OCT4a^neg^, NANOG^weak^, and SOX2^weak^). Interestingly, a recent characterization of trophoblast progenitor cells derived from first trimester placentae reveals that undifferentiated trophoblast progenitor cells that form embryoid bodies and that are capable of multipassage also display OCT4^neg^, NANOG^weak^, and SOX2^weak^ phenotypes [39]. Due to the association of this transcription marker expression profile with the presence of progenitor-like functions in HTR8/SVneo cells and in trophoblast progenitor cells, it is enticing to conclude that NANOG and SOX2 are responsible for the observed functions. Further studies with gain/loss-of-function analyses would, however, be necessary to finalize that conclusion. Furthermore, since CDX2 is coexpressed in HTR8/SVneo cells with certain hESC transcription factors, it is most plausible that the “stemness-” associated transcription factor regulatory network has been corrupted, probably through alterations secondary to transfection of HTR8 parent cells with a plasmid containing the simian virus 40 large T antigen (SV40). 

We were surprised to see that NANOG was not expressed in JEG3 cells as described in a recent publication, in which NANOG was especially detected in the nuclear fraction of JEG3 cells [40]. Currently, we cannot explain this discrepancy other than in methodology differences, and further analyses on the mRNA and protein level are under way. 

The NOTCH1 signal was remarkably visible in both cell lines. NOTCH1 has been associated with “stemness” properties, as well as with the differentiation of cancer stem cells (tumor stem-like cells) into endothelial progenitor cells [18, 19, 27]. Furthermore, NOTCH1 expression is linked with trophoblast, as well as with malignant types of invasion [17, 20, 28]. In the physiologic placenta, NOTCH1 expression is thought to be vital for placental angiogenesis, while defective NOTCH signaling is thought to contribute in the pathogenesis of preeclampsia (reviewed in [41]). With our current information, it is as yet impossible to conclude whether NOTCH1 expression is a sign of stem-like properties, of EMT or invasion potential. Further functional analyses would be helpful in unraveling this aspect.

In summary, though, all of the previously analyzed characteristics of HTR8/SVneo are in line with the idea that HTR8/SVneo cells share more similarities to a trophoblast progenitor cell, perhaps the cytotrophoblast, than a primary extravillous trophoblast. Our investigation, together with that of Takao et al., is first step in defining HTR8/SVneo progenitor cell characteristics; however, the final step of in vivo testing per xenotransplantation into immuncompromised mice is as yet pending (but seems likely to be successful, since xenografts of human placental explants into immuncompromised mouse is highly successful [42]). 

In all finality, we hypothesize that transfection of the HTR8/SVneo cell line has altered the original (extravillous) trophoblast cell in a manner leading to development of the characteristics described in these investigations; thus, investigators should use caution when using the popular HTR8/SVneo cell line as a model for primary extravillous trophoblast cells.

We wish also to direct investigators to the expression alterations (or lack thereof) seen in our immunocytochemistry and immunofluorescence results before and after blockage with human serum. Our results suggest, as with [22], that trophoblastic cells are prone to unspecific binding, and caution should be exercised when interpreting immunostaining results. 

## Figures and Tables

**Table 1:** List of antibodies.

Immunohistochemistry (IHC)				
Antibody	Clone	Isotype	Concentration IHC	Source
Cdx2	—	Polyclonal Rabbit	1 : 200	Cell Signaling
Sox2	D6D9	Polyclonal Rabbit	1 : 100	Cell Signaling
Notch1	D6F11	Polyclonal Rabbit	1 : 200	Cell Signaling
Nanog	—	Polyclonal Rabbit	1 : 400	Cell Signaling
Oct4A	C52G3	Polyclonal Rabbit	1 : 300	Cell Signaling
Isotyp control	DA1E	Polyclonal Rabbit	1 : 100	Cell Signaling
ABC Elite kit (rabbit IgG)				Vector Laboratories (Lörrach, Germany)
